# Prognostic value of variables derived from heart rate variability in patients with traumatic brain injury after decompressive surgery

**DOI:** 10.1371/journal.pone.0245792

**Published:** 2021-02-04

**Authors:** Hsueh-Yi Lu, Abel Po-Hao Huang, Lu-Ting Kuo

**Affiliations:** 1 Department of Industrial Engineering and Management, National Yunlin University of Science and Technology, Douliou, Yunlin County, Taiwan; 2 Division of Neurosurgery, Department of Surgery, National Taiwan University Hospital, Taipei, Taiwan; University of Rome 'La Sapienza', ITALY

## Abstract

Measurement of heart rate variability can reveal autonomic nervous system function. Changes in heart rate variability can be associated with disease severity, risk of complications, and prognosis. We aimed to investigate the prognostic value of heart rate variability measurements in patients with moderate-to-severe traumatic brain injury after decompression surgery. We conducted a prospective study of 80 patients with traumatic brain injury after decompression surgery using a noninvasive electrocardiography device for data collection. Assessment of heart rate variability parameters included the time and frequency domains. The correlations between heart rate variability parameters and one-year mortality and functional outcomes were analyzed. Time domain measures of heart rate variability, using the standard deviation of the RR intervals and the square root of the mean squared differences of successive RR intervals, were statistically significantly lower in the group of patients with unfavorable outcomes and those that died. In frequency domain analysis, very low-frequency and total power were significantly higher in patients with favorable functional outcomes. High-frequency, low-frequency, and total power were statistically significantly higher in patients who survived for more than one year. Multivariate analysis using a model combining age and the Glasgow Coma Scale score with variables derived from heart rate variability substantially improved the prognostic value for predicting long-term outcome. These findings reinforced the concept that traumatic brain injury impacts the brain-heart axis and cardiac autonomic modulation even after decompression surgery, and variables derived from heart rate variability may be useful predictors of outcome.

## Introduction

Traumatic brain injury (TBI) is a common cause of death and disability in adults worldwide. Moderate or severe TBI can significantly impact a patient’s life and productivity and there is a wide variation in the long-term outcomes following TBI [1–3]. Reliable assessment of prognostic factors in patients with TBI may guide appropriate treatment strategies, recovery efforts, and allocation of healthcare resources; this makes it essential to identify reliable and easy-to-measure criteria associated with long-term outcomes [4, 5]. A variety of factors associated with prognostic outcomes following TBI have been recognized in previous studies, including the Glasgow Coma Scale (GCS) score, age, pupillary light response, and the Injury Severity Score (ISS) [6–8]. However, insufficient inter- and intra-rater reliabilities in clinical settings make it difficult to rigorously evaluate the universal predictors of long-term outcome.

Heart rate variability (HRV), which is derived from electrocardiogram (ECG) findings, has been widely applied as a noninvasive and reliable technique to measure autonomic nervous system (ANS) activity and to determine the pathological and physiological associations with autonomic dysfunction in patients with various disorders, such as myocardial infarction, diabetes, trauma, adrenal insufficiency, and sepsis [9–12]. For patients with TBI, autonomic neural activity reflects the compensatory response to traumatic injury and medical treatments [13], and autonomic dysfunction may be associated with mortality and functional outcome. However, data on the correlation between autonomic dysfunction and long-term functional outcome in patients with TBI after decompression surgery in a hospital setting are lacking. This prospective study was designed to investigate the prognostic value of variables derived from HRV. The hypothesis was that HRV measured after surgery is associated with long-term functional outcome and mortality in patients with TBI. We found significant correlations between HRV measures and outcomes, with further analysis demonstrating the efficacy of these measures, in combination with the patient’s age and GCS score Scale, in predicting long-term outcomes and their potential for improving early treatment decisions and resource allocation.

## Methods

### Patients

Consecutive patients with TBI admitted to the intensive care unit (ICU) at the National Taiwan University Hospital and its Yunlin branch were screened for eligibility. This study was approved by the Committee on Human Studies at the National Taiwan University Hospital, and written consent for the publication of findings was obtained from the patients or their families. For the patients who regained consciousness and were able to communicate soon after treatment, written consent was obtained from the patients themselves. For the other patients, written consent was obtained from their families. All procedures performed were in accordance with the ethical standards of the institutional research committee and with the 1964 Helsinki Declaration and its later amendments or comparable ethical standards. The diagnosis of TBI was confirmed by brain computed tomography (CT). Only patients aged over 18 years with moderate-to-severe isolated brain injury (defined as a GCS score of 3–12) who underwent surgery within 24 hours of injury were recruited into this study. TBI mainly included the following diagnoses: subdural hematoma, epidural hematoma, subarachnoid hemorrhage, cerebral contusion, and diffuse axonal injury. The exclusion criteria were: (a) a history of preexisting brain diseases such as brain tumor, meningitis, and stroke; (b) abuse of substances such as illicit drugs and alcohol; (c) combined traumatic injuries including rib fracture, hemothorax, liver or spleen laceration, and any type of bone fracture, except skull fracture; (d) any known cardiac disease or heart failure; and (e) inability to obtain good quality ECG signals in the first 24 h after surgery. A total of 80 patients with TBI were recruited into the study from January 2013 to March 2015.

### Assessment and management

Patients were triaged on arrival to the emergency department, and the attending physician evaluated the GCS score and conducted an immediate cerebral CT scan for each patient. The patients were treated according to the Advanced Trauma Life Support guidelines and the American Association of Neurological Surgeons/Congress of Neurological Surgeons Guidelines for the Management of Head Injury.

The selection of surgical approach (i.e., craniectomy, craniotomy, and/or hematoma evacuation with intraparenchymal placement of a fiberoptic intracranial pressure monitor [Camino Model 110-4BT; Camino Laboratories, San Diego, CA, USA]) for intracranial hemorrhage or brain swelling was based on the patient’s GCS score, pupillary response, CT findings, age, and presence or absence of neurological deterioration. All patients were admitted to the ICU after surgery. Postoperative management included mechanical ventilation, head elevation (30°), fluid resuscitation, medical treatment for control of brain swelling, and nutritional support. After being transferred to the ward, patients participated in rehabilitation programs and were followed for at least 12 months postoperatively in an outpatient setting.

### Data collection

The demographic data (age and sex), GCS, and medical history (diabetes mellitus and hypertension) were documented on admission to the ICU. ECG data following surgery and the Glasgow Outcome Scale (GOS) score 12 months postoperatively were obtained from clinical records.

### ECG recording

A noninvasive cardiac monitoring device (Ez Sleep Recorder; DynaDx, Mountain View, CA, USA) was used to directly acquire and record ECG data with a sampling rate of 200 Hz. The signals were obtained by attaching surface electrodes to the chest wall, with recording taking place for a 2-hour period after the patient was admitted to the neurosurgical ICU post-operatively. The ECG recordings started at least two hours after awakening from general anesthesia. Through a USB connection, the acquired data was downloaded from the device to the computer and stored. ECG signals were then filtered and cleaned using Kubios software (University of Eastern Finland, Kuopio, Finland) [14] to remove noise artifacts (using a band pass filter of 0.5–40 Hz) and perform spectral analysis. In continuous ECG data, an R-R interval is determined as a cardiac cycle by detecting adjacent R waves within QRS complexes [15]. To obtain ECG records appropriate for HRV analysis, a five-minute segment of continuous data that was sufficiently clean to distinguish certain R-R intervals from noise and artifacts was selected for each patient.

### HRV measurement and variables

The two main analyses of HRV performed included time domain and frequency domain measurements. In the time domain analysis, two variables are calculated: standard deviation of the RR intervals (SDNN) and the square root of the mean squared differences of successive RR intervals (RMSSD). In the frequency domain analysis, which is more valid and informative over short periods of sampling, a fast Fourier transform (FFT) was performed to convert a series of R-R intervals into different spectral components of the frequency domain ranges. The equation below was used to obtain the value of the discrete Fourier transform using the FFT algorithm: [16]

$$X[k]=\sum_{k=0}^{N-1}x[n]e^{-\frac{2\pi jkn}{N}}$$

where X is the value of the discrete Fourier transform, N is the number of data points, and n and k represent indices with values ranging from 0 to N-1. The integrated area of the total power (TP, 0.01–0.6 Hz), high-frequency (HF, 0.16–0.40 Hz), as well as low frequency (LF, 0.04–0.15 Hz) bands in the absolute value of power (ms^2^), and the LF/HF ratio were calculated. To minimize the effects of the variation in power spectrum among patients, the LF and HF bands were normalized by dividing the integrated LF and HF areas by the total power area and multiplying the value by 100.

### Outcome measures

Long-term outcomes were assessed at 12 months using the GOS score [17]. The GOS is a simple five-point scale that classifies patients into one of five categories: 1, dead; 2, vegetative state; 3, severe disability; 4, moderate disability; and 5, good recovery. In our study, we developed models to predict the 12-month functional outcome and mortality. The variables for the functional outcome were coded as unfavorable (GOS score of 1–3) and favorable (GOS score of 4–5) outcomes. For the prediction of mortality, the outcome was dichotomized into dead (GOS score of 1) or alive (GOS score of 2–5).

### Statistical analysis

The data of the spectral power components were logarithmically transformed in order to diminish the impact of its non-Gaussian distribution [18]. For descriptive statistics, numerical data were expressed as mean and standard error. Normally and non-normally distributed numerical variables were analyzed with an independent samples *t*-test and the Mann-Whitney U-test, respectively. Categorical variables were analyzed using cross-table statistics (chi-square or Fisher’s exact test). Multivariate logistic regression was performed to identify power spectral components that could independently predict outcomes after controlling for age and GCS score as covariates. Two-tailed tests were used with a statistical significance level below 0.05 and 0.01. Calibration ability was determined by using Cox and Snell, and Nagelkerke R2 coefficients in the Hosmer-Lemeshow goodness-of-fit test. The discriminatory performance was assessed by receiver operating characteristic (ROC) analysis to calculate sensitivity, specificity, the Youden Index, and the area under the receiver operating characteristic curve (AUROC). The Youden Index is a value of the optimal prediction threshold that maximizes the combination of sensitivity and specificity (Youden Index = sensitivity + specificity—1) [19]. AUROC measures the two-dimensional area underneath the ROC curve, which is plotted at all classification thresholds. This shows the performance of the classification model. A value between 1 and 0.9 is considered excellent, a value between 0.9 and 0.8 is considered good, a value between 0.8 and 0.7 is considered reasonable, and values less than 0.7 are considered poor [20, 21].

## Results

Eighty patients with TBI were enrolled during the study period. The patients’ demographic and clinical data are outlined in Tables 1 and 2. The mean age was 58.5 years, 61.3% of the patients were male, 22.5% reported a history of diabetes mellitus, and 47.5% were diagnosed with hypertension. Good functional recovery was achieved in 27 (33.8%) patients, and 75% were alive at six months. In terms of functional outcome, patients with poor outcomes were significantly older (62.4 years) and had a lower GCS score before surgery (46 out of 53 had a GCS score less than score 8) than those with good outcomes (mean age of 50.9 years and 10 out of 27 had a GCS score less than 8). Patients who died within 12 months of the TBI had lower GCS scores (20 out of 20 had a GCS score less than 8) than those who survived (36 out of 60 had a GCS score less than 8).

**Table 1:** Distributions of demographic and clinical variables.

Description	Mean/Frequency	S.D./Percentage
**Age**	58.5	19.2
**Gender *(male/female)***	49/31	61.3/38.7
**Diabetes mellitus history *(yes/no)***	18/62	22.5/77.5
**Hypertension history *(yes/no)***	38/42	47.5/52.5
**GCS in ER *(≤8*, *>8)***	56/24	70.0/30.0
**6-mon functional outcome *(not good/good)***	53/27	66.3/33.8
**6-mon mortality *(dead/alive)***	20/60	25.0/75.0

ER: emergency room, GCS: Glasgow outcome scale

**Table 2:** Comparison of demographic and clinical variables in patients with TBI.

	Functional outcomes		Mortality	
	Unfavorable	Favorable	Dead	Alive
	(n = 53)	(n = 27)	(n = 20)	(n = 60)
**Age**	62.4 (17.5)^a^	50.9 (20.4)**	60.5 (21.5)	57.8 (18.5)
**Sex (male/female)**	30/23	19/8	13/7	36/24
**Diabetes mellitus history *(yes/no)***	15/38	3/24	6/14	12/48
**Hypertension history *(yes/no)***	24/29	14/13	9/11	29/31
**GCS (≤8, >8)**	46/7	10/17**	20/0	36/24^††^

*t*-test for continuous variables and chi-square test for dichotomous variables

^a^ Mean (standard error)

*p < 0.05 and

**p<0.01 significant when comparing between Unfavorable and Favorable groups

^†^p < 0.05

^††^p<0.01 significant when comparing the Dead and Alive groups

The results of the HRV analysis with respect to functional outcome (poor/good) and mortality (dead/alive) are shown in Table 3. The time domain analysis showed that the SDNN and RMSSD were significantly lower in patients with unfavorable outcomes and those that died.

**Table 3:** Comparison of heart rate variability measures in patients with TBI.

	Functional outcomes		Mortality	
Variables	Unfavorable (GOS ≤ 3)	Favorable (GOS > 3)	Dead (GOS = 1)	Alive (GOS > 1)
	**n = 53**	**n = 27**	**n = 20**	**n = 60**
**SDNN (ms)**	19.8 (2.1)	34.6 (4.6)**	13.5 (2.2)	28.6 (2.7)^††^
**RMSSD (ms)**	16.5 (2.4)	31.4 (4.7)**	9.6 (1.7)	25.5 (2.9)^††^
**HF (ms**^**2**^**)**	218.1 (74.7)	303.8 (65.3)	36.8 (13.0)	317.1 (69.9)^†^
**LF (ms**^**2**^**)**	147.3 (40.44)	224.81 (44.6)	52.1 (18.4)	213.9 (39.7)^†^
**VLF (ms**^**2**^**)**	202.8 (37.6)	785.1 (316.3)*	153.4 (47.1)	481.3 (148.2)
**TP (ms**^**2**^**)**	569.3 (125.7)	1315.9 (349.1)*	242.5 (66.5)	1014.2 (190.5)^†^
**LF/HF**	2.2 (0.5)	1.8 (0.4)	2.4 (1.1)	1.9 (0.3)
**Ln(HF)**	3.2 (0.3)	4.8 (0.3)**	2.1 (0.5)	4.3 (0.2)^††^
**Ln(LF)**	3.2 (0.3)	4.7 (0.3)**	1.9 (0.6)	4.3 (0.2)^††^
**Ln(VLF)**	4.3 (0.3)	5.7 (0.3)**	3.6 (0.5)	5.1 (0.2)^††^
**Ln(TP)**	5.2 (0.2)	6.5 (0.2)**	4.4 (0.4)	6.1 (0.2)^††^
**Ln(LF/HF)**	1.2 (0.2)	1.1 (0.1)	1.3 (0.5)	1.1 (0.1)

*p < 0.05 and

**p<0.01 significant when comparing between Unfavorable and Favorable groups

^†^p < 0.05

^††^p<0.01 significant when comparing the Dead and Alive groups

Values are means (standard error)

In the frequency domain analysis, patients with favorable functional outcomes had significantly higher VLF power (785.1 vs. 202.8) and TP (1315.9 vs. 569.3). HF power (317.1 vs. 36.8), LF power (213.9 vs. 52.1), and TP (1014.2 vs. 242.5) were significantly higher in patients who were alive compared with those who died within one year after TBI. When spectral components were logarithmically transformed to confirm a normal distribution, significant decreases in spectral power were found in the majority of frequency domain indices such as Ln(HF), Ln(LF), Ln(VLF), and Ln(TP) for patients in the unfavorable and dead groups.

With regard to prediction of functional outcome (unfavorable/favorable), two multiple logistic regression models were constructed to compare the predictive ability of variables derived from HRV (Table 4). The first model included only age and GCS score as variables, and the second included age, GCS score, SDNN, RMSSD, TP, VLF, LF, and HF. Since other clinical variables, such as sex, diabetes mellitus history, and hypertension history, were not found to be significantly associated with functional outcome and mortality in our univariate analyses, they were not included. When compared to the first model, the Hosmer-Lemeshow goodness-of-fit test revealed that the second model combining age and GCS score with the HRV variables had substantially improved calibration, in which Cox and Snell R2 was increased from 0.32 to 0.42, and Nagelkerke R2 increased from 0.45 to 0.58. The second model also demonstrated an excellent improvement in discrimination, with sensitivity values ranging from 0.77 to 0.89 and AUROC values ranging from 0.81 to 0.91. Two comparison models were developed for the prediction of mortality, and similar results were found in terms of calibration and discrimination abilities. The values of Cox and Snell R2 (from 0.20 to 0.33), Nagelkerke R2 (from 0.29 to 0.48), sensitivity (from 0.55 to 0.76), Youden index (from 0.40 to 0.56), and AUROC (from 0.74 to 0.86) were all increased markedly from the first to the second model (Table 4).

**Table 4:** Performance of the long-term outcome prediction models.

	Calibration (HL test)		Discrimination			
Models	C & S R^2^	N R^2^	Sen	Spec	Youden	AUROC
**Predict Unfavorable/Favorable**						
** Age, GCS ≦ 8**	0.32	0.45	0.77	0.76	0.53	0.81
** Age, GCS ≦ 8, SDNN, RMSSD, Ln(TP), Ln(VLF), Ln(LF), Ln(HF)**	0.42	0.58	0.89	0.78	0.67	0.91
**Predict Dead/Alive**						
** Age, GCS ≦ 8**	0.20	0.29	0.55	0.85	0.40	0.74
** Age, GCS ≦ 8, SDNN, RMSSD, Ln(TP), Ln(VLF), Ln(LF), Ln(HF)**	0.33	0.48	0.76	0.82	0.56	0.86

HL test: Hosmer-Lemeshow goodness of fit test; C & S R^2^: Cox and Snell R^2^; N R^2^: Nagelkerke R^2^; Sen: Sensitivity; Spec: specificity; AUROC: area under receiver operating characteristic curve

## Discussion

The ANS plays a critical role in maintaining cardiovascular homeostasis and regulating the total peripheral resistance to blood flow through the vagal cholinergic and sympathetic noradrenergic nerves. HRV is a marker of the sympathetic-parasympathetic balance of the ANS [22]. The interaction between the brain and heart is well-understood [23, 24]. Acute stroke has been reported to affect cardiac autonomic responses, and the proposed mechanisms include changes in the hypothalamic-pituitary-adrenal axis activity, balance of the sympathetic and parasympathetic systems, catecholamine surge, immunodepression, and inflammation [25]. An increasing number of studies report that the quantitative autonomic response, determined through HRV analysis, could provide insight into the outcomes following TBI [26–28]. For example, one study used prehospital ECG data to analyze the association of HRV with in-hospital mortality after injury. The results showed that patients who died from their injury had lower normalized LF power, higher HF power, and higher HF-to-LF ratios [13]. Rapenne et al. reported that HRV was significantly correlated with imminent brain death in patients with severe head injury and suggested that it could act as an early indicator [18].

This study compared the clinical characteristics and HRV findings between post-operative TBI patients with unfavorable and favorable functional outcomes, and between patients who survived and did not survive for one year after surgery. It is the first to analyze the feasibility and predictive value of HRV within 24 h after decompression surgery for TBI. Our results demonstrate that several HRV indices are independent predictors of poor functional outcome and mortality, and suggest that noninvasive HRV indices such as SDNN and RMSSD, and normalized indices such as Ln(HF), Ln(LF), Ln(VLP), and Ln(TP), are more sensitive than conventional scores or indices, such as GCS score and, age at predicting the long-term outcome of surgical patients with TBI.

Regarding the time domain analysis of HRV, SDNN and RMSSD were significantly reduced in the subgroup with unfavorable outcome/mortality within 12 months compared to the favorable outcome/survival groups.

In short-term five-minute SDNN recordings, the primary source of variation is parasympathetically mediated respiratory sinus arrhythmia. RMSSD is influenced by parasympathetic activity to a greater degree than SDNN [29]. SDNN and RMSSD were significantly lower in patients with TBI, ischemic stroke, and other acquired brain injuries [30–32]. In the frequency domain analysis, a significant decrease in Ln(HF), Ln(LF), Ln(VLF), and Ln(TP) was observed in subgroups with unfavorable outcome and mortality. These results indicate that reduced sympathetic and parasympathetic activation in response to TBI after decompression surgery is a predictor of unfavorable outcome. The HF component represents ANS parasympathetic activity and respiratory sinus arrhythmia, which is the oscillation of activity as a result of the frequency of respiration. The LF component represents the interaction between the sympathetic and parasympathetic nervous systems. LF/HF, the ratio of LF to HF power, indicates the balance between sympathetic and parasympathetic effects on cardiac autonomic function [33]. The VLF (0.0033–0.04 Hz) component is mainly dependent on parasympathetic tone and has been proposed to as an indicator of neuroendocrine and thermoregulatory effects on the heart [34]. The spectral components were logarithmically transformed to confirm their normal distribution in our analysis, which could support the true significance of these variables.

The spectral components were logarithmically transformed to confirm the normal distribution and to adjust for the skewness of the distribution when the analysis was performed, instead of presenting the absolute units (ms^2^); this could also support the true significance of these variables [35, 36].

Studies have shown a reduction in some HRV indices in patients with acute stroke, which can predict post-stroke mortality or poor outcome [33, 37, 38]. Reductions in the VLF and LF components have been found to be correlated with outcomes in critically ill neurosurgical patients [39]. Biswas et al. reported that the LF/HF ratios decreased significantly in the presence of an intracranial pressure > 30 mmHg or cerebral perfusion pressure < 40 mmHg in children with TBI (n = 15), and that higher LF/HF ratios can predict favorable outcomes [26]. LF, HF, and LF/HF have been reported to be prognostic predictors in TBI (n = 19) [28, 40]. The VLF and LF components have been reported to be lower in comatose patients (n = 16) after TBI than in healthy controls [41]. These results could be explained by direct physical damage or the indirect effect of increased intracranial pressure on the control centers of the ANS, including the insula, cingulate gyrus, amygdala, hypothalamus, and brainstem.

The present study has several limitations. The duration of HRV acquisition for analysis was limited to five minutes from the two-hour recordings, which could be interpreted as a methodological limitation of our study. Although we started to record ECG data two hours after the surgery, the effects of general anesthesia and peri-operative drugs may influence the HRV analysis. Another limitation was the small sample size. However, our study focused only on patients with moderate to severe TBI who underwent surgical treatment. In addition, HRV may be confounded by factors such as age, mechanical ventilation, beta-blockers, diabetes mellitus, and sedatives. Future studies in larger populations with specific medical and pre-operative conditions may further establish the predictive value of HRV in patients with TBI.

## Conclusions

Our study demonstrated that multiple HRV measurements were significantly correlated with 12-month functional outcome and mortality in patients with moderate-to-severe TBI who underwent surgery. In addition to age and GCS score, these HRV variables helped to construct predictive models with an AUROC of 0.91 and 0.86 for functional outcome and mortality, respectively. These findings reinforce the concept that TBI has a great impact on the brain-heart axis and cardiac autonomic modulation, and that HRV variables may be useful predictors of outcome.
